# Who hit the ball out? An egocentric temporal order bias

**DOI:** 10.1126/sciadv.aav5698

**Published:** 2019-04-24

**Authors:** Ty Y. Tang, Michael K. McBeath

**Affiliations:** Department of Psychology, Arizona State University, Tempe, AZ, USA.

## Abstract

Temporal order judgments can require integration of self-generated action events and external sensory information. We examined whether conscious experience is biased to perceive one’s own action events to occur before simultaneous external events, such as deciding whether you or your opponent last touched a basketball heading out of bounds. Participants made temporal order judgments comparing their own touch to another participant’s touch, a mechanical touch, or an auditory click. In all three manipulations, we find a robust bias to perceive self-generated action events to occur about 50 ms before external sensory events. We denote this bias to perceive self-actions earlier as the “egocentric temporal order” bias. Thus, if two players hit a ball nearly simultaneously, then both will likely have different subjective experiences of who touched last, leading to arguments.

## Teaser

Self-generated action events are perceived to occur before external events, leading to differences in subjective temporal order.

## INTRODUCTION

In everyday life, we must temporally integrate our own motor action events with externally generated sensory information. This integration becomes particularly important in fast-paced sports such as basketball and soccer, where players frequently make judgments concerning the order of nearly simultaneous events. Oftentimes, disagreements in these judgments can lead to heated arguments and hostility between opponents. Typically, these perceptual disagreements appear to arise from either random errors in temporal order judgments or players trying to deceptively exploit temporally ambiguous situations. The present study investigates the alternative that a systematic bias exists to perceive self-generated action events as having occurred before simultaneous external events.

A wealth of research has shown that perception and action are inextricably linked, with the experience of perception frequently occurring through self-action (*1*–*3*). In particular, research in intentional binding has investigated the relationship between voluntary action and temporal perception. Intentional binding occurs when a voluntary action and a delayed action event are temporally coupled and the experienced delay between the two is subsequently diminished. This phenomenon is specific to operant actions, and the effect is not present for involuntary movements (*4*). Other studies have replicated the initial findings of Haggard *et al*. (*4*), in addition to establishing the link between intentional binding and the sensation of agency (*5*–*8*). The intentional binding literature includes two findings of particular interest to the present study. In 2012, Haering and Kiesel (*9*) found that when presenting participants with two simultaneous stimuli, if they were told one of the two stimuli was caused by their previous action, they perceived that action event as having occurred before the other event. Capozzi *et al*. (*10*) also found results showing that when judging sequential action events, participants perceived self-generated and externally generated events to be temporally repulsed from one another. In contrast to real-world events, however, intentional binding research necessitates an artificial delay inserted between the action and action event of its experimental paradigm, as follows:

Stimulus ➔ Action ➔ Delay ➔ Action event ➔ Temporal order judgment

**Fig. 1:**
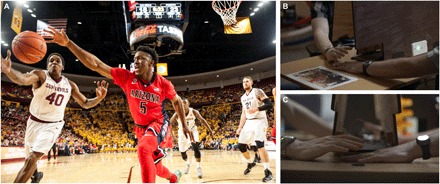
The real-world case and experimental proxy. (**A**) Two basketball players reaching toward the ball and nearly simultaneously knocking it out of bounds. (**B**) The experimental setup, with two participants sitting across a table, ready to tap one another’s hand. The green light cues participants when to touch. (**C**) Close-up on the participants’ hands. The participant on the left is preparing to tap the participant on the right. Photo credits: (A) J and L Photography, Getty Images Sport and (B and C) Robert Ewing, Arizona State University.

This delay, usually in the range of several tenths of a second, is not typically observed when interacting with the physical world. When making temporal order judgments in sports and athletics, such as in Fig. 1A, voluntary actions and action effects are temporally continuous, with no unnatural delay between the end of the action and the onset of the action event.

## METHODS AND RESULTS

**Fig. 2:**
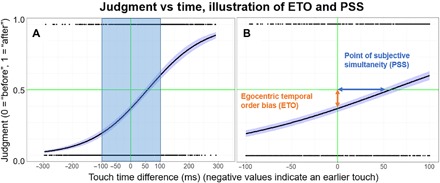
Visual demonstration of ETO and PSS offsets. The black line indicates an individual’s probability of thinking they touched before or after the other participant, as a function of the time difference between the touches. The vertical green line indicates the time at which both touches from both participants were truly simultaneous; the horizontal green line indicates where participants are equally likely to respond “I touched first” or “they touched first.” The black dots on the bottom and top of each graph correspond to individual responses, coded I touched first and they touched first, respectively. (**A**) Plot of the predicted results, assuming participants perceive their own action events as occurring first. (**B**) A zoom in on the blue area of (A). The PSS is represented by the horizontal time offset from the origin, and the ETO bias is represented by the vertical judgment offset from the origin.

To investigate whether there is a systematic bias to perceive one’s own action events as having occurred before external events in real-world temporal order judgments, we had Arizona State University (ASU) student participants (ages 18 to 23) play a timing game. In our first experiment, as shown in Fig. 1 (B and C), pairs of student participants sat across from each other at a table with a divider between them, removing any visual information regarding the other participant. Sixteen undergraduate students (11 females and 5 males) with normal or corrected-to-normal vision and normal touch perception were cued by a randomly timed flash of light to touch a capacitive sensor on the back of the other person’s left hand using their right index finger. Both participants then made anonymous temporal order judgments about who had touched the other first by manually tapping one of two buttons. Using a natural tactile versus tactile stimulus comparison, we eliminated any temporal perception differences due to potential differences in processing rate between different senses (*11*) or differences in temporal resolution between different senses (*12*, *13*). Each pair of participants made 50 judgments. No feedback of actual time differences or response accuracy was given to participants, they were not allowed to communicate with their partner, and correct responses were not incentivized. An illustrative model of theoretical results is shown in Fig. 2 to clarify term definitions.

**Fig. 3:**
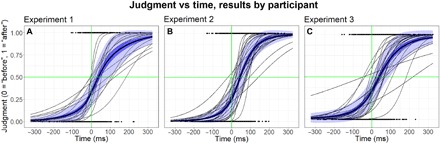
Individual and overall average results. The thin black lines represent each participant’s psychometric curves, and the bold black line indicates the mean across participants. The dark blue ribbon represents one SE, and light blue ribbon represents one SD. The black dots on the bottom and top of each graph correspond to individual responses, coded “I touched before” = 0 and “I touched after” = 1, respectively. (**A**) Experiment 1, two participants tap one another on the hand. (**B**) Experiment 2, one participant compared his/her touch time against a mechanical solenoid’s touch. (**C**) Experiment 3, one participant compared his/her touch time against an auditory click.

Figure 3A shows the results of the first experiment. Fitting a logistic binary regression model to the data reveals a significant, large effect size bias for participants to believe that they touched their partner first. The analysis reveals an egocentric temporal order (ETO) bias indicating a 67% probability of reporting “I touched first” at a 0-ms offset, (*t*_15_ = 17.40, *P* < 0.001, *d* = 4.10). This ETO corresponds with an actual time delay for which participants perceived that both touches were simultaneous (denoted the “point of subjective simultaneity” or PSS) was a positive offset of 51.2 ms (*t*_16_ = 3.88, *P* < 0.001, *d* = 0.91). This PSS indicates that, on average, when participants touched their partner 51.2 ms after their partner touched them, they perceived the events as simultaneous. Another way to interpret the PSS is that participants systematically perceived their own action event as having occurred 51.2 ms earlier than a simultaneous external event.

In our second experiment, we replaced the “partner” with a fast-acting solenoid to deliver the tactile stimulus. This allowed us to investigate whether the ETO bias was a socially induced phenomenon due to interacting with another person. Twenty-five undergraduate ASU participants (18 females and 7 males) made 50 temporal order judgments each between their own touch and the mechanical tactile stimulus. Participants were again cued with a light stimulus. In a replication of our first experiment, we again found a significant main effect bias for participants to believe that their touch occurred before a simultaneous mechanical tactile stimuli, with an ETO bias indicating a 75% chance of reporting “I touched first” at a 0-ms offset (*t*_24_ = 23.47, *P* < 0.001, *d* = 4.69) and a corresponding PSS of 47.8 ms (*t*_24_ = 6.56, *P* < 0.001, *d* = 1.31) (Fig. 3B).

In our final experiment, 25 ASU undergraduate participants (17 females and 8 males) made 50 temporal order judgments each between their own touch and an auditory click following a light flash. As in the previous experiments, we found the same bias even when participants performed this cross-sensory integration of their own tactile action event and an external auditory stimuli (ETO bias: 67% chance of responding “I touched first” at 0-ms offset, *t*_24_ = 20.22, *P* < 0.001, *d* = 4.56; PSS: 45.3 ms, *t*_24_ = 3.77, *P* < 0.001, *d* = 0.75) (Fig. 3C).

In a post hoc analysis of data, there were no significant correlations between the ETO bias and demographic variables such as sex, handedness, or arm length. The lack of a handedness difference served as a control against a potential dominant hand effect.

## CONCLUSION

Across all three experiments, we confirmed a significant bias, with large effect sizes, that participants judge action events generated by their own voluntary actions to occur about 50 ms before simultaneous externally generated events. A between-subjects analysis of variance of all three experiments reveals no significant difference between the ETO bias [*F*_2,64_ = 2.15, not significant (n.s.)] or the PSS (*F*_2,64_ = 0.071, n.s.), with the measured variance in both cases captured primarily by the residuals (93.71% for ETO bias and 99.77% for PSS). We suggest that the ETO bias is consistent with a constructive, predictive model of perception, wherein the temporal offset is caused by a delayed integration of externally generated perceived events into our stream of consciously experienced action (*14*). This model proposes that anticipated self-generated perception actions are perceived in near “real time,” which allows for rapid, predictive, real-time behaviors such as precisely hitting a moving ball. In contrast, nonanticipated events are perceptually delayed by about 50 ms, which is consistent with the well-established temporal resolution of visual flicker and auditory fusion rates around 20 Hz (*15*). This model is also supported by findings from the intentional binding literature that disruption of the presupplementary motor area, a brain region associated with prediction, attenuates the temporal binding of the action event (*16*), and can explain phenomena such as the perceived temporal reorganization of events associated with predictable sensory outcomes (*17*, *18*). If the ETO bias is a product of a constructive, predictive internal model, then differences may also be seen as a function of athletic expertise, as studies have shown that expert athletes demonstrate enhanced action anticipation, although how these differences might affect the ETO bias remains to be seen (*19*, *20*). We suggest that the ETO bias may also be moderated by the supplementary motor complex, an area shown to activate differently for self-initiated actions versus externally triggered actions (*21*). In addition, evidence showing that our own “self-actions” are perceptually attenuated relative to the environment could be coupled with a reliance on internal feed-forward comparator models for judging voluntary action events compared to external perceptual cues (*22*).

## DISCUSSION

The results presented here contribute to our understanding of temporal perception and the importance of vantage on perception and can help account for why sports calls and judgments can become so heated and divisive (*11*). Our principal finding, showing that individuals have a robust bias to judge self-generated action events as having occurred about 50 ms earlier, potentially sheds light on arguments that arise in sports over temporal order judgments. The results also support evidence that differing vantage points and locus of control play a major role in perception and action. Our findings can help facilitate understanding in accepting differences between subjective experiences. They also offer insight into solutions for computerized, objective temporal judges and contribute to a larger understanding of human temporal perception and biases. Briefly, we have identified what may be a principal cause of arguments in ballgames, and it is about time.

## Supplementary Material

http://advances.sciencemag.org/cgi/content/full/5/4/eaav5698/DC1

Download PDF

Data file S1

## SUPPLEMENTARY MATERIALS

Supplementary material for this article is available at http://advances.sciencemag.org/cgi/content/full/5/4/eaav5698/DC1 Data file S1. Raw data file.
