# Supplementary material for: Who hit the ball out? An egocentric temporal order bias
Source: Sci Adv. 2019 Apr 24;5(4):eaav5698. doi: 10.1126/sciadv.aav5698 (PMC6482011; doi:10.1126/sciadv.aav5698)
Supplement: http://advances.sciencemag.org/cgi/content/full/5/4/eaav5698/DC1 [file supp_5_4_eaav5698__index.html]

Science Advances | Science Advances

## Supplementary Materials

Download PDF

**Other Supplementary Material for this manuscript includes the following:**

- Data file S1 (Microsoft Excel format). Raw data file.

**Files in this Data Supplement:**

- Adobe PDF - aav5698\_SM.pdf
