# Supplementary material for: Who hit the ball out? An egocentric temporal order bias
Source: Sci Adv. 2019 Apr 24;5(4):eaav5698. doi: 10.1126/sciadv.aav5698 (PMC6482011; doi:10.1126/sciadv.aav5698)
Supplement: Download PDF [file aav5698_SM.pdf]

Science Advances
AAAS

advances.sciencemag.org/cgi/content/full/5/4/eaav5698/DC1

Supplementary Materials for

Who hit the ball out? An egocentric temporal order bias

Ty Y. Tang* and Michael K. McBeath

*Corresponding author. Email: tytang24@asu.edu

Published 24 April 2019, *Sci. Adv.* **5**, eaav5698 (2019)
DOI: 10.1126/sciadv.aav5698

Other Supplementary Material for this manuscript includes the following:

(available at advances.sciencemag.org/cgi/content/full/5/4/eaav5698/DC1)

Data file S1 (Microsoft Excel format). Raw data file.
